# P70 Adult sepsis treatment: optimizing antibiotic prescribing using clinical and microbiological factors—a systematic review

**DOI:** 10.1093/jacamr/dlag102.076

**Published:** 2026-06-26

**Authors:** Abeeda Farooq, Rasha Abdelsalam Elshenawy

**Affiliations:** School of Health, Medicine and Life Sciences, University of Hertfordshire, College Lane, Hatfield AL10 9AB, UK; School of Health, Medicine and Life Sciences, University of Hertfordshire, College Lane, Hatfield AL10 9AB, UK

## Abstract

**Background:**

Sepsis is a life-threatening organ dysfunction caused by a dysregulated host response to infection, leading to organ failure and death if untreated.^1^ Globally, 48.9 million cases occur annually, causing 11 million deaths, representing 20% of all global mortality, with the greatest burden in low- and middle-income countries (LMICs).^2^ Antimicrobial resistance (AMR) further complicates treatment, as inappropriate antibiotic selection drives resistance and worsens outcomes.^3^ Prescribing variation across settings remains substantial, highlighting the need to identify variables that reliably inform antibiotic decision-making in adult sepsis.

**Objectives:**

To identify clinical, physiological and laboratory variables associated with antibiotic selection and outcomes in adult sepsis; to map regimens to the WHO AWaRe classification; and to compare prescribing patterns across ICU and non-ICU settings in HICs and LMICs.

**Methods:**

A systematic review was conducted following PRISMA 2020 guidelines. Five databases were searched (MEDLINE via PubMed, Embase, Web of Science, CINAHL and Cochrane Library) from 2016 to 2026 across three domains: population (sepsis, septic shock, bacteraemia), severity variables (SOFA, qSOFA, APACHE II, NEWS2, biomarkers) and treatment (antibiotics, empirical therapy, de-escalation, AWaRe). PICO principles guided study selection. Eligible studies included observational, randomized controlled and diagnostic studies in adults aged 18 or over with confirmed or suspected sepsis. Data were extracted into a standardized form and analysed using quantitative frequency analysis, with antibiotic regimens mapped to WHO AWaRe categories. No ethics approval was required as no primary data were collected.

**Results:**

Across 228 records identified, 13 studies were included, with sample sizes ranging from 103 to 60 507 participants, spanning both high-income countries (HICs) and low- and middle-income countries (LMICs) across four continents. The SOFA score was the most reliable severity indicator, demonstrating the highest sensitivity for predicting mortality. Although qSOFA showed high specificity, its sensitivity was critically low in LMIC settings (54.3%), meaning it missed more than half of sepsis cases. Procalcitonin (PCT) was the strongest biomarker for detecting bacteraemia and supported safe antibiotic de-escalation, reducing treatment duration by six days without increasing mortality (8 versus 14 days). However, PCT was unavailable in most LMICs. Delays in antibiotic administration beyond 12 h independently increased 30 day mortality. Antibiogram-guided therapy reduced mortality from 81.8% to 18.2% compared with empirical-only prescribing, while full guideline adherence reduced mortality from 11.3% to 6.4% and shortened hospital stay by two days. Watch-category antibiotics predominated globally. MDR rates were alarmingly high in LMICs, including 100% MDR isolates in Malawi and 57% carbapenem-resistant organisms in India, rendering empirical Watch antibiotics largely ineffective.

**Conclusions:**

This study demonstrates that sepsis mortality is directly linked to the speed and accuracy of antibiotic decision-making. Antibiogram-guided therapy and SOFA-based assessment reduced mortality dramatically. However, prescribing dominated by Watch-class antibiotics continues to fail in LMICs where MDR rates render standard regimens ineffective. PCT-guided stewardship safely shortens treatment duration without compromising outcomes. The evidence is clear: integrating clinical, microbiological and physiological variables into prescribing decisions is not optional; it is lifesaving. Context-specific antibiotic frameworks are urgently needed globally to improve treatment appropriateness, combat AMR and close the sepsis mortality gap.
